# Enhancing Patient Understanding of Laboratory Test Results: Systematic Review of Presentation Formats and Their Impact on Perception, Decision, Action, and Memory

**DOI:** 10.2196/53993

**Published:** 2024-08-12

**Authors:** Frederieke A M van der Mee, Fleur Schaper, Jesse Jansen, Judith A P Bons, Steven J R Meex, Jochen W L Cals

**Affiliations:** 1 Department of Family Medicine Care and Public Health Research Institute Maastricht University Maastricht Netherlands; 2 Department of Clinical Chemistry Reinier Medical Diagnostic Center Delft Netherlands; 3 Central Diagnostic Laboratory Maastricht University Medical Center+ Maastricht Netherlands

**Keywords:** electronic health record, patient access to records, patient portal, laboratory test results, clinical laboratory information systems, health communication, health informatics, patient engagement, patient involvement

## Abstract

**Background:**

Direct access of patients to their web-based patient portal, including laboratory test results, has become increasingly common. Numeric laboratory results can be challenging to interpret for patients, which may lead to anxiety, confusion, and unnecessary doctor consultations. Laboratory results can be presented in different formats, but there is limited evidence regarding how these presentation formats impact patients’ processing of the information.

**Objective:**

This study aims to synthesize the evidence on effective formats for presenting numeric laboratory test results with a focus on outcomes related to patients’ information processing, including affective perception, perceived magnitude, cognitive perception, perception of communication, decision, action, and memory.

**Methods:**

The search was conducted in 3 databases (PubMed, Web of Science, and Embase) from inception until May 31, 2023. We included quantitative, qualitative, and mixed methods articles describing or comparing formats for presenting diagnostic laboratory test results to patients. Two reviewers independently extracted and synthesized the characteristics of the articles and presentation formats used. The quality of the included articles was assessed by 2 independent reviewers using the Mixed Methods Appraisal Tool.

**Results:**

A total of 18 studies were included, which were heterogeneous in terms of study design and primary outcomes used. The quality of the articles ranged from poor to excellent. Most studies (n=16, 89%) used mock test results. The most frequently used presentation formats were numerical values with reference ranges (n=12), horizontal line bars with colored blocks (n=12), or a combination of horizontal line bars with numerical values (n=8). All studies examined perception as an outcome, while action and memory were studied in 1 and 3 articles, respectively. In general, participants’ satisfaction and usability were the highest when test results were presented using horizontal line bars with colored blocks. Adding reference ranges or personalized information (eg, goal ranges) further increased participants’ perception. Additionally, horizontal line bars significantly decreased participants’ tendency to search for information or to contact their physician, compared with numerical values with reference ranges.

**Conclusions:**

In this review, we synthesized available evidence on effective presentation formats for laboratory test results. The use of horizontal line bars with reference ranges or personalized goal ranges increased participants’ cognitive perception and perception of communication while decreasing participants’ tendency to contact their physicians. Action and memory were less frequently studied, so no conclusion could be drawn about a single preferred format regarding these outcomes. Therefore, the use of horizontal line bars with reference ranges or personalized goal ranges is recommended to enhance patients’ information processing of laboratory test results. Further research should focus on real-life settings and diverse presentation formats in combination with outcomes related to patients’ information processing.

## Introduction

An increasing number of patients have direct access to their own web-based patient portal. This includes diagnostic test results ordered by their health care professional, such as laboratory test results [1,2]. Providing patients with web-based access to patient portals aims to enhance patient involvement in their health management. Improving patients’ knowledge and self-efficacy may enhance disease self-management and interactions with health care providers, and ultimately lead to better health outcomes and increased satisfaction with care [3-6].

However, patient access to web-based patient portals also has potentially negative consequences. For example, misinterpretation or inaccurate knowledge could lead to underestimation of test results and promote a false sense of security [7]. Similarly, gaining insight into medical test results might trigger feelings of insecurity, anxiety, and confusion [8-12]. Previous studies have shown that poor understanding of test results can lead to an increase in telephone calls or doctor consultations, emergency department visits, and even hospitalizations [13-15]. As a result, the overall utility or benefit of providing lab results directly to patients may depend on how these data are presented to and interpreted by the patient [16,17].

Limited health literacy and numeracy skills are significant barriers to the effective use of web-based patient portals and understanding of laboratory test results [18,19]. Although patient understanding can be improved to some extent by avoiding medical jargon and using plain language, overcoming the problem of incomprehension in its entirety remains an ongoing challenge [19-21]. One of the key issues is the numerical presentation of test results, especially for patients with low numeracy skills (ie, those with limited ability to derive meaning from numbers), who have been shown to have difficulties in interpreting basic laboratory test results and identifying results that fall outside the reference range [18]. The lack of supporting information and guidance on interpretation of results contributes to the problem of misinterpretation. This challenge becomes even more pronounced when a larger number of test results are presented [18].

Basic patient portals typically present laboratory test results in a numerical format, often accompanied by a reference range (ie, the range that represents normal values for a particular test) [10,22,23]. An alternative approach to communicating test results is the use of visual displays, such as colors or graphics. These formats require less health literacy and numeracy skills for interpretation and may improve patients’ understanding of the results [24-28]. Previous studies have examined a variety of presentation formats for communicating laboratory test results. However, direct comparisons between these studies can be challenging due to the variety of presentation options and clinical contexts. In addition, not all formats may be appropriate for every clinical situation [29].

There is only limited evidence on the effect of specific presentation formats on patients’ information processing. As highlighted by Witteman and Zikmund-Fisher [17], laboratory test results often lack meaning for the patients receiving them. Test results represent data, which differs from information and actual knowledge patients commonly encounter in daily life [30,31]. Patients have to complete several steps to go from data perception to usable knowledge. Ancker et al [32] described these steps as well, based on the Wickens model of human information processing [33]. In a sequential order, patients need perception and behavioral intention to achieve actual health behavior. Therefore, it is important that these separate steps of information processing are taken into account when presentation formats are evaluated.

Our systematic review aims to synthesize the existing evidence on effective components of presentation formats for laboratory test results focusing on patients’ perception, decision, action, and memory. In this review, we will specifically focus on numeric laboratory test results, and not on results containing only textual or nonnumeric findings.

## Methods

This review was reported in accordance with the PRISMA (Preferred Reporting Items for Systematic Reviews and Meta-Analyses; Multimedia Appendix 1) [34]. A protocol for this review was not previously registered.

### Search Strategy

The search was conducted in 3 databases (PubMed, Web of Science, and Embase) from inception up to May 31, 2023. In each database, a search was performed, which was developed by the first author (FM) together with an experienced librarian and contained both thesaurus and free text terms. For the search in Embase, a filter was applied to remove preprint records and to exclude MEDLINE citations, since the latter were already covered by the PubMed search. Additionally, 2 authors (FM and FS) performed backward snowballing by screening reference sections of all selected articles to identify relevant publications missed with the search strategy. A fully reproducible search can be found in Multimedia Appendix 2.

### Study Selection and Eligibility Criteria

All identified titles and abstracts were downloaded to reference management software (Endnote) and duplicates were removed. Two authors (FM and FS) independently screened for potential eligible articles using Covidence, a Cochrane’s technology platform [35]. First, titles and abstracts were screened against the eligibility criteria. Second, full texts of potentially suitable articles were rescreened using the same criteria. In case of disagreement, consensus was reached by discussion or screening by a third reviewer (JC).

We considered articles fitting for inclusion if they were original research. Studies describing or comparing different ways of presenting diagnostic laboratory test results to patients were included. Only studies examining numeric laboratory test results were included. Furthermore, studies are needed to evaluate the effect of communicating test results on patients’ comprehensibility, attitudes, or experiences. Studies conducted in primary care and secondary/tertiary care settings were eligible, as well as studies including healthy volunteers. Studies had to be written in English or Dutch.

Studies were excluded if they (1) were protocols, reviews, systematic reviews, meta-analyses, book chapters, editorials, letters, practice pointers, oral presentations, or poster presentations; (2) were about development, implementation, or adoption of web-based patient portals in general, or about the type of notification of laboratory test results, if they did not consider patients’ interpretation of the lab results; (3) focused on web-based access to notes, and not to laboratory test results; (4) did not mention type of presentation format of lab results; (5) focused on the development of web-based lifestyle interventions or web-based applications to collect patient-reported outcomes; (6) focused on the safety or privacy issues of web-based patient portals; (7) were about the effect of communicating test results in web-based patient portals on patients’ medication management; (8) tested the effect of test result communication on health care providers; (9) examined communication of other types of diagnostic test results (eg, pharmacogenomics or genomics, radiology, pathology, or microbiology); and (10) examined communication of test results in the context of screening programs.

### Data Extraction

Two authors (FM and FS) independently extracted data from the eligible studies into a prepared spreadsheet. The spreadsheet was developed by the multidisciplinary team and piloted by both authors. For each study, the year of publication, country in which the study was performed, study design, number of participants, description of the study population, and the inclusion and exclusion criteria were assessed. Furthermore, information about the presentation of test results in the portal, the type of laboratory tests studied, and whether real or mock data were used, was extracted.

### Outcome Measures

Previous research regarding this subject focused on a variety of outcomes related to patients’ information processing. As stated above, Ancker et al [32] introduced a taxonomy to categorize different outcome measures when communicating numbers in health care. These categories include sequentially; perception, decision/behavioral intention, action/actual health behavior, and memory. Perception is further divided into 4 subcategories: affective perception, perceived magnitude, cognitive perception, and perception of communication [32,36,37]. An explanation of the categorized outcome measures can be found in Textbox 1 [32]. For this review, the outcome measures of each study were extracted and classified into the categories described.

**Explanation of the outcome measure categories based on patients’ information processing by Ancker et al.**

**Affective perception**

- Feelings about the laboratory result communicated.

**Perceived magnitude**

- Perceived size of risk associated with a test result, captured with measures as “how large or small does this value seem to you?”

**Cognitive perception**

- Understanding whether a laboratory result is elevated, normal, or below normal. Being able to identify direction of a trend over time.

**Perception of communication**

- Preference for presentation format of test result.

**Decision**

- Intention to seek more information or to change behavior after viewing results.

**Action**

- Change in actual health behavior (eg, search for more information).

**Memory**

- Recall of a specific test result after viewing (ie, verbatim recall).

### Quality Assessment

To assess the quality and risk of bias of all included studies, the Mixed Methods Appraisal Tool (MMAT) was used [38]. The MMAT is designed to concomitantly appraise studies with different designs, such as qualitative, quantitative, and mixed methods studies [39]. Question sets are specific to the study design, notably qualitative studies, quantitative randomized controlled trials, quantitative nonrandomized studies, quantitative descriptive studies, and mixed methods studies. For each suitable study, the appropriate category was chosen and the criteria stated for this specific category were rated as “yes,” “no,” or “can’t tell.”

Two authors (FM and FS) discussed both data and quality extraction until a consensus was reached.

### Data Synthesis

Due to the heterogeneity of study designs and primary outcomes, meta-analysis was considered inappropriate. Instead, narrative synthesis was used to integrate the findings into descriptive summaries regarding ways of presenting laboratory test results and outcomes of interest.

## Results

### Overview

**Figure 1:**
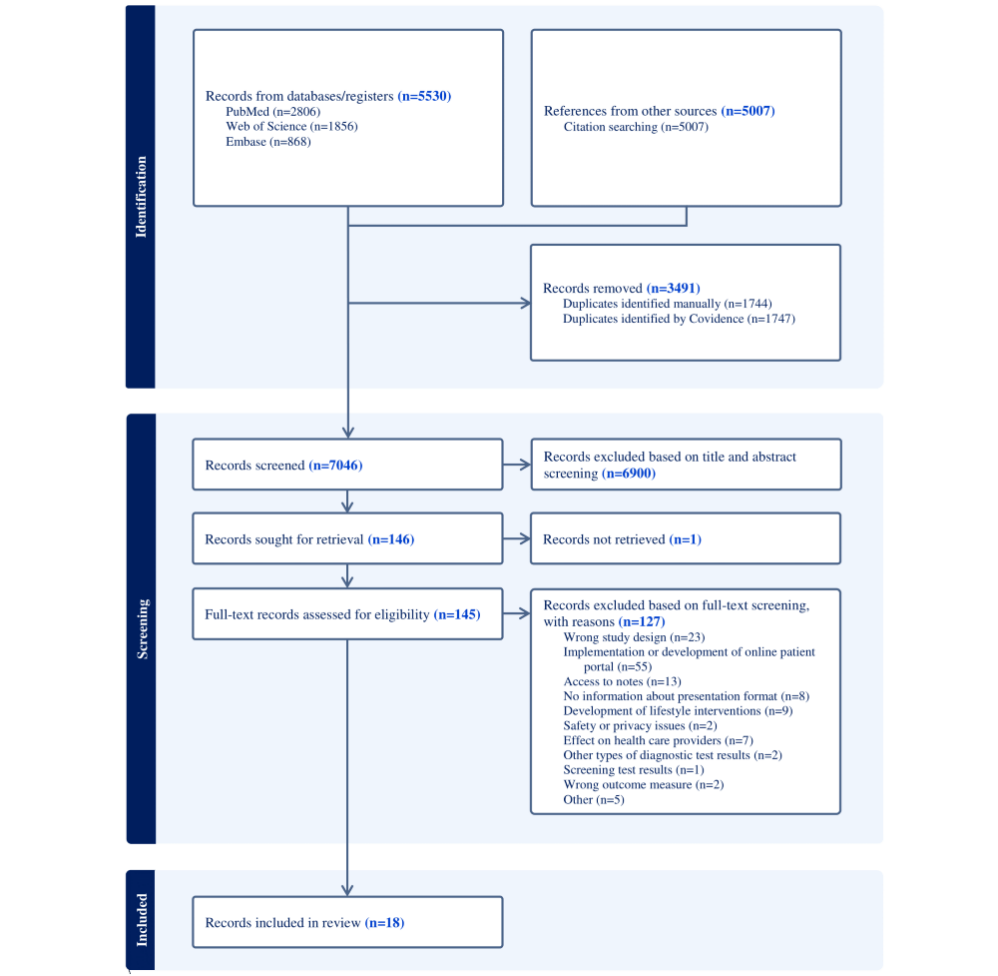
Flowchart of the study selection process.

The initial search identified 10,537 references. A total of 3490 duplicate records were removed. After applying the exclusion criteria in the primary title and abstract screening, another 6900 records were removed. During full-text screening of the remaining articles (n=146), it appeared that 1 full text was not available. Furthermore, 127 articles were excluded because they did not meet the eligibility criteria. Describing the implementation of web-based patient portals, unrelated to laboratory test results, was the most common exclusion criterion (55/127, 43.3%; Figure 1). A total of 18 studies were found eligible for this systematic review. Cohen κ for interrater reliability was 0.62 for title and abstract screening and 0.80 for full-text screening, indicating respectively a moderate and strong agreement between the 2 reviewers [40].

### Study Characteristics

A total of 2 qualitative studies, 11 quantitative studies, and 5 mixed methods studies were included (n=18). The included studies were published between 2012 and 2021, and the majority were conducted in the United States (n=13, 72%). The total sample size of the included studies was 12,225 participants, ranging from 8 to 6766 participants. Among the articles reporting the following characteristics, sex was almost equally distributed (6219/13,155, 47.3% female), and participants were predominantly middle-aged (mean 51.1 years) and White (8429/10,865, 77.6% on average). Fourteen (78%) of the 18 studies reported educational level, with 48% (5676/11,813) of the participants reporting a higher education (defined as college-degree or higher). Overall characteristics of the included studies and populations are summarized in Table 1.

**Table 1:** Study and population characteristics of all included studies (n=18).

Author (year)	Country	Study design	Sample (n)	Population characteristics						Aim of study
				Sample	Sex (% female)	Mean age in years (SD or range)	Race and ethnicity	Education^a^		
Bar-Lev et al (2020) [41]	Israel	Survey	225	Convenience sample	55.9	35 (14)	—^b^	0% low education, 32.6%, middle education, 61.6% high education, and 5.8% other	To examine how different visual displays of personalized medical information affect laypersons’ understanding, perceptions, and actions	
Brewer et al (2012) [42]	United States	Randomized controlled trial; nonrandomized experimental study	106	Convenience sample	79.2	46 (30-83)	82% White	0% low education, 27% middle education, and 73% high education	To compare the relative usability of tables and horizontal bar graphs for presenting medical test results electronically to consumers	
Elder et al (2012) [43]	United States	Qualitative study	12	Convenience sample	67	60 (34-73)	83% White, 8% Black, and 8% Asian	0% low education, 58% middle education, and 42% high education	To understand patients’ experiences with, and preferences for, results notification and communication in primary care settings	
Fraccaro et al (2018) [7]	United Kingdom	Nonrandomized experimental study	20	Real patients	20	51.8 (10.3)	—	5% low education, 35% middle education, and 60% high education	To investigate if presentations using color improve patients’ interpretation of laboratory test results presented through patient portals	
Hohenstein et al (2018) [44]	United States	Mixed methods study	301	Volunteers	51	46.0 (16.3, 18-90)	66.8% White, 19.6% Hispanic/ Latino/ Spanish, 12.3% Black/ African American/ Negro, and 4% Asian	0% low education, 38.2% middle education, 48.2% high education, 13.6% unknown	To explore how people interpret medical test results, examined in various interface designs developed to enable self-care and health management	
Kelman et al (2016) [45]	United States	Survey	211	Convenience sample	90	52.7 (10.0)	89% White, 4% African American, 6% other and 0.5% preferred not to answer	0.5% low education, 57% middle education, 41% high education, and 1% unknown	To explore ways in which laboratory test results can be communicated in a patient-friendly manner	
Morrow et al (2017)^c^ [46]	United States	Mixed methods study	36	—	67	77 (65-89)	—	—	A pilot study to finalize development of video-enhanced messages before conducting formal evaluation studies	
Morrow et al (2019)^c^ [47]	United States	Randomized controlled trial	144	—	71.5	71.9 (60-94)	—	18.8% low education, 13.2% middle education, and 68% high education	To investigate how to support older adult comprehension of and response to patient portal-based numerical information	
Nystrom et al (2018) [48]	United States	Mixed methods study	14	Real patients	—	43 (25-73)	—	—	To study patient’s ability to generate meaning from each test result and how this meaning would inform their decision-making and subsequent actions	
Scherer et al (2018) [49]	United States	Randomized controlled trial	6766	Mixed sample	50.9	49.1 (15.8)	78.2% White, 14.8% African America, and 9.7% other	2% low education, 52.2% middle education, and 45.8% high education	To test the impact of including clinically appropriate goal ranges outside the standard range in the visual displays of laboratory test results	
Struikman et al (2020) [50]	The Netherlands	Randomized controlled trial	487	Volunteers	50.3	52.8 (15.4)	—	7.7% low education, 45.8% middle education, 46.4% high education	To discover whether the way of presenting blood test outcomes in an electronic patient portal is associated with patient health engagement and whether this varies across different test outcomes	
Talboom-Kamp et al (2020) [51]	The Netherlands	Survey	354	Real patients	—	—	—	—	To investigate attitudes, experiences, and self-efficacy of patients using an online patient portal that communicates laboratory test results	
Tao et al (2018) [23]	China	Nonrandomized experimental study	72	Convenience sample	56	Young adult group: 22.3 (2.6); older adult group: 65.8 (3.6)	—	1.4% low education, 33.3% middle education, and 65.3% high education	To examine the effects of 4 graphical formats and age on consumers’ comprehension, perceptions, visual attention, and preference of the graphs of the use of self-monitoring test results	
Zarcadoolas et al (2013) [52]	United States	Qualitative study	28	Volunteers	64.3	40.0 (12.4, 21-63)	25% Hispanic, 3.6% non-Hispanic White, 67.9% non-Hispanic Black, and 3.6% other	46.4% low education, 53.6% middle education, and 0% high education	To identify vulnerable consumers’ response to patient portals, their perceived utility and value, as well as their reactions to specific portal functions	
Zhang et al (2020)^c^ [15]	United States	Mixed methods study	203	Volunteers	48.3	63.5 between 26-49 years	69.5% White, 4.4% Asian or Pacific islander, 16.7% African American, 5.9% Hispanic or Latino, 2% American Indian, and 1.5% other	0% low education, 19.7% middle education, 79.9% high education, and 0.4% other	To examine the challenges and needs of patients when comprehending laboratory test results	
Zhang et al (2021)^c^ [53]	United States	Mixed methods study	8	—	50	18-64	—	—	To examine how to help patients understand the connections between their medical context and test results, and the necessary support and actions after receiving these test results	
Zikmund-Fisher et al (2017)^d^[22]	United States	Survey	1620	Volunteers	52.3	48.9 (15.7)	77.4% White, 13% African American, and 7% other	1.9% low education, 49.9% middle education, and 48.2% high education	To investigate the extent to which different visual displays help people discriminate between test results that do or do not require urgent action	
Zikmund-Fisher et al (2018)^d^ [54]	United States	Randomized controlled trial	1618	Volunteers	52.1	48.8 (19-89)	77.8% White, 13.2% Black, 13.2% Hispanic, 4% Asian, 0.8% native American, and 4.3% other or multirace	0% low education, 0% middle education, 50.1% high education, and 49.9% unknown	To test the effect of including an additional harm anchor reference point in visual displays of laboratory test results	

^a^Low education: primary school. Middle education: secondary, high, or trade school or some college. High education: 4-year, college, associate, university, undergraduate, bachelor’s, master’s, advanced, professional, or doctorate degree.

^b^Not available.

^c^The following articles are pilot and main studies: Morrow et al (2017) [46] and (2019) [47], as well as Zhang et al (2020) [15] and (2021) [53].

^d^The following articles originate from the same parent study: Zikmund-Fisher et al (2017) [22] and (2018) [54].

**Figure 2:**
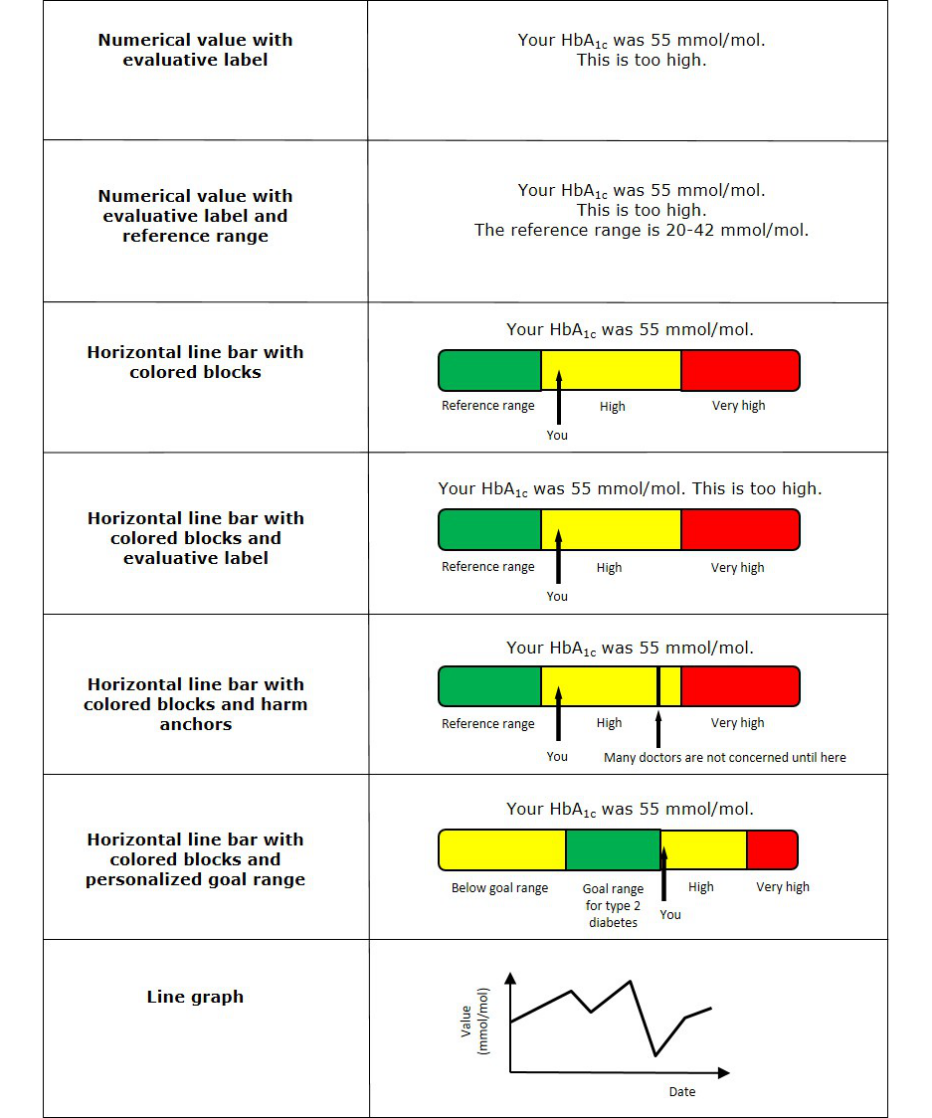
Examples of presentation formats used for displaying laboratory test results. The examples are based on a hypothetical HbA_1c_ test result. A combination of different presentation formats is possible. HbA_1c_: hemoglobin A_1c_.

The most frequently used laboratory tests were lipid profile (n=10) and hemoglobin A_1c_ (HbA_1c_) or glucose (n=5). In total, 4 studies used real patients as study population, other studies used healthy volunteers, a convenience sample, or a mixed sample (n=12) or did not define their study population (n=3). Studies used mock test results (ie, hypothetical results; n=16), real results (n=1, with real patients), or both (n=1). The majority of studies used numerical values with reference ranges (n=12) or horizontal line bars with colored blocks (n=12; Table 2). A more detailed overview of the different ways of presenting test results is provided in Multimedia Appendix 3 [7,15,22,23,41-54]. An explanation of the different presentation formats can be found in Figure 2.

**Table 2:** Laboratory test characteristics and presentation format used in all included studies (n=18).

Author (year)	Laboratory test information and presentation format						
	Laboratory test	Type of data	Presentation format				
			Numerical	Horizontal line bar	Graph	Video	Text only
Bar-Lev et al (2020) [41]	Hemoglobin, cholesterol, progesterone	Mock	✓		✓		✓
Brewer et al (2012) [42]	Total cholesterol, HDL^a^, LDL^b^	Mock	✓	✓			
Elder et al (2012) [43]	Total cholesterol, HDL, LDL	Mock	✓	✓	✓		✓
Fraccaro et al (2018) [7]	Creatinine, eGFR^c^, potassium	Mock	✓	✓			
Hohenstein et al (2018) [44]	Vitamin B12, procalcitonin, cholesterol	Mock	✓	✓			
Kelman et al (2016) [45]	Rheumatoid factor	Mock	✓				
Morrow et al (2017) [46]	Total cholesterol, HDL, LDL, TG^d^, HbA_1c_^e^	Mock				✓	
Morrow et al (2019) [47]	Total cholesterol, HDL, LDL, TG, HbA_1c_	Mock	✓	✓		✓	
Nystrom et al (2018) [48]	Total cholesterol, HDL, LDL, TG	Mock		✓			
Scherer et al (2018) [49]	HbA_1c_	Mock	✓	✓			
Struikman et al (2020) [50]	Hemoglobin, TSH^f^, vitamin D	Mock	✓	✓			
Talboom-Kamp et al (2020) [51]	Type of test differed per patient	Real		✓			
Tao et al (2018) [23]	Glucose (fasting and postprandial)	Mock		✓			
Zarcadoolas et al (2013) [52]	Total cholesterol, HDL, LDL, TG, HbA_1c_	Mock	✓				
Zhang et al (2020) [15]	Total cholesterol, HDL, LDL, TG	Real and mock	✓				
Zhang et al (2021) [53]	Total cholesterol, HDL, LDL	Mock		✓			
Zikmund-Fisher et al (2017) [22]	Platelet count, ALT^g^, creatinine	Mock	✓	✓			
Zikmund-Fisher et al (2018) [54]	Platelet count, ALT, creatinine	Mock		✓			

^a^HDL: high-density lipoprotein.

^b^LDL: low-density lipoprotein.

^c^eGFR: estimated glomerular filtration rate.

^d^TG: triglycerides.

^e^HbA_1c_: hemoglobin A1c.

^f^TSH: thyroid stimulating hormone.

^g^ALT: alanine aminotransferase.

### Quality Assessment

The quality assessment tool (MMAT) includes 5 assessment criteria per study design, each of which is given a score of 20% if present (Multimedia Appendix 4 [7,15,22,23,41-54]). Both qualitative articles (n=2) scored 100%, indicating excellent quality. Quantitative articles (n=11) scored between 0% and 100%, indicating a broad range of quality. These articles lost points mainly for sampling issues (biased sampling strategies and unrepresentative samples), randomization issues (unclear randomization process and incomparable groups at baseline), unclear blinding process, and lack of clarity about the completeness of outcome data and nonresponse bias. Mixed methods articles (n=5) scored between 60% and 100% (low-to-high quality), for the same reasons as described above. In addition, weaknesses in these articles included having an unclear rationale for using a mixed methods design, unclear presentation format, and failure to adequately interpret the results of the integration of qualitative and quantitative findings.

### Outcome Measures

#### Overview

In all 18 studies, perception was an outcome measure, further categorized into affective perception (n=7), perceived magnitude (n=6), cognitive perception (n=10), and perception of communication (n=14; Table 3 and Textbox 1). Additionally, 10 studies assessed behavioral intention, while memory was considered as an outcome measure in 3 of the included studies.

**Table 3:** The outcomes assessed in all included studies (n=18).

Author (year)	Perception					Decision		Action		Memory
	Affective perception	Perceived magnitude	Cognitive perception	Perception of communication	Behavioral intention		Health behavior		Verbatim recall	
Bar-Lev et al (2020) [41]		✓			✓					
Brewer et al (2012) [42]				✓					✓	
Elder et al (2012) [43]			✓	✓						
Fraccaro et al (2018) [7]		✓			✓				✓	
Hohenstein et al (2018) [44]	✓		✓	✓						
Kelman et al (2016) [45]			✓	✓	✓					
Morrow et al (2017) [46]	✓		✓	✓						
Morrow et al (2019) [47]	✓	✓		✓	✓				✓	
Nystrom et al (2018) [48]				✓	✓					
Scherer et al (2018) [49]	✓		✓		✓					
Struikman et al (2020) [50]	✓		✓		✓					
Talboom-Kamp et al (2020) [51]	✓			✓	✓					
Tao et al (2018) [23]		✓	✓	✓						
Zarcadoolas et al (2013) [52]			✓	✓						
Zhang et al (2020) [15]	✓		✓	✓			✓			
Zhang et al (2021) [53]			✓	✓						
Zikmund-Fisher et al (2017) [22]		✓		✓	✓					
Zikmund-Fisher et al (2018) [54]		✓		✓	✓					

#### Affective Perception

Several studies explored participants’ confidence and concerns while viewing and interpreting laboratory results [15,44,47,49,51]. Talboom-Kamp et al [51] demonstrated that presenting laboratory test results in horizontal line bar format with colored blocks and evaluative labels (ie, textual explanation) enhanced participants confidence in managing their own health, although this effect was not significant. No comparison between different presentation formats and the influence on confidence was described. These comparisons were also lacking in the other studies.

When results were presented in a horizontal line bar format with colored blocks and a personalized goal range, the negative affect was significantly higher than when results were presented without colored blocks [49].

Scherer et al [49] studied the use of personalized reference values or goal ranges. A type 2 diabetes mellitus scenario was studied, in which standard reference ranges are often not applicable. Replacing standard ranges with goal ranges significantly reduced perceived discouragement compared with situations without goal display, highlighting a positive effect of goal ranges on affective perception [49]. Furthermore, 2 other studies recommended the use of personalized reference ranges in their discussion [44,51].

In 3 studies, whether laboratory test results were within reference ranges seemed to be more important than the presentation format. As results moved further from the reference range, positive emotions decreased and negative emotions increased [15,46,47]. This change in affective perception was not influenced by message format.

#### Perceived Magnitude

The perceived magnitude of risk of extremely out-of-range results remained unaffected by the presentation formats in all studies. However, for near-normal or slightly out-of-range results participants encountered difficulties in estimating test result severity. Accurate risk perception was lacking, since the severity of these results was inconsistently overestimated or underestimated [7,22,41,47,54]. Zikmund-Fisher et al [54] demonstrated that the incorporation of harm anchors (ie, a threshold line outside the reference range labeled “many doctors are not concerned until here”) significantly enhanced adequate estimations of test result severity for slightly out-of-range results.

Three studies investigated the effect of presentation format on the perceived size of risk [22,23,47]. Morrow et al [47] compared horizontal line bars with both numerical and video-enhanced formats. For both low- and borderline-risk scenarios, the perceived magnitude of risk was significantly higher when horizontal line bars were used, indicating that participants tend to overestimate risk for normal results [47]. Tao et al [23] did not specify whether result normality affected risk perception using different types of horizontal line bars. However, when personalized information was added to the line bar, the risk was perceived as significantly higher. Interestingly, despite this, participants expressed a preference for personalized line bars [23]. Zikmund-Fisher et al [22] compared different types of horizontal line bars with a numerical format. Participants expressed the highest risk perception when near-normal results were presented in a numerical format with a reference range, whereas the perceived risk was lowest when horizontal line bars with gradient colors were used [22].

#### Cognitive Perception

In all 10 studies assessing this outcome, participants consistently demonstrated the ability to understand or identify out-of-range results. There was consensus among these studies that presenting numbers with a reference range only was insufficient and that tailored information was needed [45,52,53]. A qualitative study revealed that participants preferred the inclusion of evaluative labels [43]. In 2 studies using horizontal line bars as the presentation format, the understanding was significantly increased when color, text, or personalized information (eg, goal range) was added [23,49].

#### Perception of Communication

The majority of included studies observed a significant association between presentation format, participant satisfaction, and ease of use. In general, satisfaction and ease of use were rated higher when test results were presented using horizontal line bars with colored blocks, as compared with other presentation formats [22,23,42,43,47,51,53]. In one qualitative study, numerical presentation with reference ranges was deemed insufficient, while graphs were considered too complex for easy comprehension [43]. Both quantitative and qualitative studies demonstrated that adding evaluative labels, such as explanations about the meaning and normality of test results, and background information about testing, enhanced understanding and effective use of results. Particularly, the use of lay terms played an important role [15,23,44,45,48,51-53]. Furthermore, 2 studies found a significant positive effect on participant satisfaction when personalized information or goal ranges were incorporated [23,51]. This addition was also recommended by 2 qualitative studies [43,53]. Zikmund-Fisher et al [54] specifically studied different types of horizontal line bars and showed no significant differences in participants’ preferences among the studied formats.

#### Decision

The behavioral intention was assessed in 10 studies, with varying focuses among them. Some authors examined whether participants would contact their physician [7,22,48,49,54], while others inquired about participants seeking additional web-based information [41,45,48], or making lifestyle changes after reviewing lab results [47,48,51].

Two studies demonstrated that the presentation format did not significantly influence participants’ need to contact their health care provider [7,49]. Conversely, Zikmund-Fisher et al [22,54] demonstrated in 2 studies that participants who viewed near-normal results in a numerical format were significantly more likely to contact their doctor compared with those viewing the same results in one of the horizontal line formats. The use of harm anchors in horizontal line bars substantially reduced the number of participants who would want to contact their physician [22,54].

Participants’ tendency to seek web-based information was significantly influenced by the presentation format, with a significantly higher inclination observed for the numerical format compared with the textual format [41]. Kelman et al [45] and Nystrom et al [48] similarly found that approximately half of the participants would look for additional information after receiving test results in numerical format with reference ranges and evaluative labels, or horizontal line bars with colored blocks, respectively. However, no comparison was made between presentation formats in these studies [45,48].

Intention to make lifestyle changes after viewing laboratory results was mentioned as an outcome in 3 studies [47,48,51]. Only one of these studies compared several presentation formats but found no significant differences between using a numerical format, horizontal line bars with colored blocks, or video-enhanced format in terms of health-beneficial intentions [47].

#### Action

There was limited data concerning the actions patients take to comprehend their test results. One mixed methods study used a numerical format with reference ranges as a presentation format [15]. Participants with abnormal test results were significantly more likely to take action compared with those with normal test results. As no comparison between presentation formats was investigated, the effect of format on action remains unstudied.

#### Memory

Variation in the presentation format of test results, using either a numerical format with reference ranges and evaluative labels, horizontal line bars with colored blocks, video presentation, or grouped presentation, did not significantly impact participant recall [7,42,47]. However, one study found a small but statistically significant effect of test result normality on memory [47].

Struikman et al [50] looked at patient health engagement (PHE), a composite measure comprising affective perception, cognitive perception, and behavioral intention. When test results were presented with explanatory text and visualization, PHE was significantly higher compared with when no explanatory information was provided [50].

## Discussion

### Principal Findings

Based on reviewing 18 articles assessing various presentation formats of laboratory test results, we can conclude there is not only one optimal presentation format in terms of patients’ perception, decision, action, and memory. Nevertheless, the results do indicate that presentation format is important for patients’ information processing.

Presentation formats differed between articles, but numerical values with reference ranges or horizontal line bars with colored blocks were most commonly used. All included studies investigated perception as an outcome measure, most frequently perception of communication (n=14). Patients’ cognitive perception and perception of communication improved when results were presented using horizontal line bars accompanied with colored blocks and evaluative labels or textual information. Incorporation of reference ranges or personalized goal ranges further enhanced patients’ perception levels. Using horizontal line bars with harm anchors significantly reduced the number of participants who would want to contact their physician compared with using a numerical format. Furthermore, using the numerical format significantly increased participants’ tendency to search for web-based information, compared with a textual format. Therefore, although no specific format is dissuaded in the included studies, the results suggest that presenting only numbers with reference ranges is suboptimal. Furthermore, adding too many colors and other information to test results could lead to an overload of visual information for some patients, and therefore ultimately decrease the amount of usable knowledge [49]. Action and memory were less frequently studied, respectively in 1 and 3 studies. Action was studied in a descriptive study not comparing different presentation formats, while memory was not significantly impacted by presentation format.

Several studies highlighted that patients’ affective perception, action, and memory were not only influenced by presentation format, but also by whether test results were within or outside the reference range. Presentation format appeared to be secondary to test result normality if results were extremely out-of-range. Nevertheless, when results were near-normal, presentation format was more important than result normality regarding effects on patients’ information processing.

Overall, the results of this review indicate that presentation format affects patients’ information processing, especially in the case of normal or near-normal test results.

### Strengths and Limitations

A multidisciplinary team of general practitioners, behavioral scientists, and clinical chemists was involved in this review, which is one of its strengths. Both presentation formats and outcomes used in the included studies were standardized by the authors using a published taxonomy to enable comparison of different studies. As the results of our review are narrative, there is a potential risk of bias when describing them, introduced by the authors. Furthermore, selection bias arising from the heterogeneity of studies represents a notable limitation of this review.

A limitation of the included studies is the use of volunteers or participants recruited via convenience sampling. Only 3 out of 18 studies used real patients, of which one study used real test results. Sixteen studies used mock test results. Displaying mock data is common practice in system evaluation. This method involves less burden and privacy risks for participants, as no personal medical data are collected. Nonetheless, participants lack personal relevance of test results when hypothetical scenarios are used. Therefore, it is possible that most of the included studies did not reflect how participants would respond in real life to their own personal health information. This may limit the generalizability of the findings. However, using personal test results could have negatively affected the comparability between studies, as each participant would have encountered different data.

Among the articles reporting educational level, 48% (5676/11,813) of the participants reported a higher education level, which is higher than in the general population. This may limit the generalizability of the findings to the overall population. Another limitation is the study heterogeneity. Included articles varied widely in methods, presentation formats, and outcome measures used. Comparison of presentation formats is challenging, especially since laboratory test result communication can have a wide range of possible purposes, from interpreting one single value to identifying important trends on time [24]. Therefore, useful presentation formats may vary per clinical scenario, which presents new challenges for designing a preferred format.

As stated above, patients have to complete several steps to go from data perception to usable knowledge [17,32]. The majority of the included studies studied the first 2 steps of this taxonomy, perception and decision. Only one study examined action as outcome measure, and 3 studies obtained information about memory. Therefore, little is known about the impact of presentation formats on actual health behavior and usable knowledge.

### Comparison With Prior Work

An increasing number of patients can directly access their laboratory test results via web; thus, it is becoming more important to make the available data meaningful to laypeople [55]. As highlighted in this review, presentation format affects patients’ information processing as described above. In cognitive science, this principle is generally known as information evaluability, in other words using relevant contextual reference information to make it easier to evaluate the meaning of in this case numerical laboratory test results (eg, is this test result good or bad, is it normal or abnormal) [56,57]. The presentation formats for laboratory test results as studied in this review could be considered as different forms of contextual information, or evaluative categories [58]. Prior research has shown that these evaluative categories add both affective and cognitive meaning to numeric test results. This enhances patients’ information processing by adding meaning and evaluability to numeric information [58-60]. Furthermore, our findings are in line with recommendations made by Witteman and Zikmund-Fisher [17]. The authors formulated 10 recommendations to communicate laboratory test results via web-based portals in ways that support understanding and actionable knowledge for patients. Our findings align with several of their recommendations, such as the importance of providing a clear takeaway message for each result, establishing thresholds for concern and action whenever feasible, and personalizing the frame of reference by permitting custom reference ranges.

This review explored different strategies to improve patients’ interpretation and comprehension of their laboratory test results. The included studies predominantly focused on the effect of graphical presentation only including a subset of the available visualization options. Other formats such as clocks or pie charts been shown in the broader numeracy literature to improve cognitive outcomes and could be the focus of further research in the context of communicating laboratory test results [61]. Graphical presentation formats might mitigate the effects of low numeracy. However, it is important to acknowledge that graphical information may not be automatically useful for individuals with limited graph literacy [62]. Besides numeracy and graph literacy, other factors such as age, educational level, health literacy, and statistical literacy (eg, understanding of concepts of uncertainty and chance) also influence patients’ information processing of such graphical results [61-63]. If one of these factors causes patients to not completely understand a specific presentation format, they may consider this format as not suitable. Therefore, some patients may require extra instructions to be able to adequately process and interpret graphical presentation formats [61]. For that reason, the interaction between patients’ literacy, numeracy, age, and educational level should be taken into account when performing future work around test result interpretation.

Several initiatives aim to inform and educate patients about laboratory test results while incorporating the insights described above. One example is Lab Tests Online, a website that provides patients with general information about laboratory tests and their meaning [64]. Recently, the usability of ChatGPT (ie, an upcoming tool based on natural language processing) to interpret laboratory test results were examined [65]. ChatGPT appeared to provide somewhat superficial interpretations, which were not always correct, and is therefore not yet usable as a primary information source for patients. However, this may change in the future with the further development of these types of tools. While our review focused on different presentation formats of laboratory test results, interpretative comments provided by laboratory specialists were not studied. Laboratory specialists often add comments to test results to assist general practitioners [66,67]. A pilot study by Verboeket-van de Venne et al [68] demonstrated a positive impact on patient empowerment when patients had access to these patient-specific comments. Therefore, further research should explore the impact of adding interpretative comments to laboratory test results on patients’ information processing.

Patients now have web-based access to not only their laboratory test results but also to medical imaging and microbiology results. Given the variations in these types of diagnostic test results, further research is warranted to explore effective components for communicating these other types of test results to patients in their web-based patient portal.

### Conclusions

As patients increasingly receive their diagnostic laboratory test results via web-based patient portals, it is becoming more and more important to make test results meaningful to them. Unnecessary confusion or anxiety should be avoided, especially when test results are outside of the reference range. The data from our systematic review suggest that horizontal line bars with colored blocks and reference ranges or personalized goal range increase patients’ cognitive perception and perception of communication. Furthermore, this format might reduce patients’ concerns and their tendency to contact their physicians. Therefore, to improve patients’ understanding of near-normal laboratory test results and prevent anxiety and concerns after viewing these results, implementing horizontal line bars with colored blocks and reference ranges or personalized goal ranges in web-based patient portals would be a prudent choice. Our review highlights the importance of taking end users (ie, patients) into consideration when designing new presentation formats. These results can guide the development and improvement of (new) web-based patient portals. Nevertheless, there is a need for further research that involves more comprehensive data collection and reporting, as well as more systematic evaluation methods. By using these findings, further research could inform the development of an interpretation support tool for laboratory test results.

## Appendix

Multimedia Appendix 1 PRISMA (Preferred Reporting Items for Systematic Reviews and Meta-Analyses) 2020 checklist.

Multimedia Appendix 2 Search strategy for PubMed, Web of Science, and Embase up to May 31, 2023.

Multimedia Appendix 3 Detailed overview of laboratory test results presentation formats in all included studies (n=18).

Multimedia Appendix 4 Detailed overview of quality assessment of all included studies (n=18).

## Abbreviations

HbA1c: hemoglobin A1c
MMAT: Mixed Methods Appraisal Tool
PRISMA: Preferred Reporting Items for Systematic Reviews and Meta-Analyses
