# Supplementary material for: Enhancing Patient Understanding of Laboratory Test Results: Systematic Review of Presentation Formats and Their Impact on Perception, Decision, Action, and Memory
Source: J Med Internet Res. 2024 Aug 12;26:e53993. doi: 10.2196/53993 (PMC11347896; doi:10.2196/53993)
Supplement: Multimedia Appendix 2 [file jmir_v26i1e53993_app2.docx]

| **Search strategy in PubMed (2,806 hits)** | | |
| --- | --- | --- |
| Search #1 | "clinical laboratory techniques"[MeSH Terms] OR "clinical laboratory services"[MeSH Terms] OR "clinical laboratory service*"[Title/Abstract] OR "laboratory"[Title/Abstract] OR "laboratory test*"[Title/Abstract] OR "blood test*"[Title/Abstract] OR "test*"[Title/Abstract] | Result # 6,469,860 |
| Search #2 | "result*"[Title/Abstract] OR "outcome*"[Title/Abstract] OR "report*"[Title/Abstract] | Result # 15,245,574 |
| Search #3 | "electronic health records"[MeSH Terms] OR "health records, personal"[MeSH Terms] OR "medical records systems, computerized"[MeSH Terms] OR "health record*"[Title/Abstract] OR "electronic health record*"[Title/Abstract] OR "personal health record*"[Title/Abstract] OR "online health record*"[Title/Abstract] OR "patient portal*"[Title/Abstract] OR "electronic patient portal*"[Title/Abstract] OR "online patient portal*"[Title/Abstract] OR "patient access to records"[Title/Abstract] OR "computerized medical record*"[Title/Abstract] | Result # 70,631 |
| Search #4 | "patient education as topic"[MeSH Terms] OR "health literacy"[MeSH Terms] OR "patient participation"[MeSH Terms] OR "self-efficacy"[MeSH Terms] OR "patient education"[Title/Abstract] OR "education of patients"[Title/Abstract] OR "health literacy"[Title/Abstract] OR "health engagement"[Title/Abstract] OR "patient participation"[Title/Abstract] OR "patients participation"[Title/Abstract] OR "patient involvement"[Title/Abstract] OR "patients involvement"[Title/Abstract] OR "patient activation"[Title/Abstract] OR "patients activation"[Title/Abstract] OR "patient engagement"[Title/Abstract] OR "patients engagement"[Title/Abstract] OR "patient empowerment"[Title/Abstract] OR "patients empowerment"[Title/Abstract] OR "patient comprehens*"[Title/Abstract] OR "patients comprehens*"[Title/Abstract] OR "patient satisfaction"[Title/Abstract] OR "patients satisfaction"[Title/Abstract] OR "experience*"[Title/Abstract] OR "understand*"[Title/Abstract] OR "interpret*"[Title/Abstract] OR "self-efficacy"[Title/Abstract] | Result # 3,346,032 |
| Search #5 | **Search #1 AND Search #2**  ("clinical laboratory techniques"[MeSH Terms] OR "clinical laboratory services"[MeSH Terms] OR "clinical laboratory service*"[Title/Abstract] OR "laboratory"[Title/Abstract] OR "laboratory test*"[Title/Abstract] OR "blood test*"[Title/Abstract] OR "test*"[Title/Abstract]) AND ("result*"[Title/Abstract] OR "outcome*"[Title/Abstract] OR "report*"[Title/Abstract]) | Result # 3,751,743 |
| Search #6 | **Search #5 AND Search #3**  ("clinical laboratory techniques"[MeSH Terms] OR "clinical laboratory services"[MeSH Terms] OR "clinical laboratory service*"[Title/Abstract] OR "laboratory"[Title/Abstract] OR "laboratory test*"[Title/Abstract] OR "blood test*"[Title/Abstract] OR "test*"[Title/Abstract]) AND ("result*"[Title/Abstract] OR "outcome*"[Title/Abstract] OR "report*"[Title/Abstract]) AND ("electronic health records"[MeSH Terms] OR "health records, personal"[MeSH Terms] OR "medical records systems, computerized"[MeSH Terms] OR "health record*"[Title/Abstract] OR "electronic health record*"[Title/Abstract] OR "personal health record*"[Title/Abstract] OR "online health record*"[Title/Abstract] OR "patient portal*"[Title/Abstract] OR "electronic patient portal*"[Title/Abstract] OR "online patient portal*"[Title/Abstract] OR "patient access to records"[Title/Abstract] OR "computerized medical record*"[Title/Abstract]) | Result # 10,910 |
| Search #7 | **Search #6 AND Search #4**  ("clinical laboratory techniques"[MeSH Terms] OR "clinical laboratory services"[MeSH Terms] OR "clinical laboratory service*"[Title/Abstract] OR "laboratory"[Title/Abstract] OR "laboratory test*"[Title/Abstract] OR "blood test*"[Title/Abstract] OR "test*"[Title/Abstract]) AND ("result*"[Title/Abstract] OR "outcome*"[Title/Abstract] OR "report*"[Title/Abstract]) AND ("electronic health records"[MeSH Terms] OR "health records, personal"[MeSH Terms] OR "medical records systems, computerized"[MeSH Terms] OR "health record*"[Title/Abstract] OR "electronic health record*"[Title/Abstract] OR "personal health record*"[Title/Abstract] OR "online health record*"[Title/Abstract] OR "patient portal*"[Title/Abstract] OR "electronic patient portal*"[Title/Abstract] OR "online patient portal*"[Title/Abstract] OR "patient access to records"[Title/Abstract] OR "computerized medical record*"[Title/Abstract]) AND ("patient education as topic"[MeSH Terms] OR "health literacy"[MeSH Terms] OR "patient participation"[MeSH Terms] OR "self-efficacy"[MeSH Terms] OR "patient education"[Title/Abstract] OR "education of patients"[Title/Abstract] OR "health literacy"[Title/Abstract] OR "health engagement"[Title/Abstract] OR "patient participation"[Title/Abstract] OR "patients participation"[Title/Abstract] OR "patient involvement"[Title/Abstract] OR "patients involvement"[Title/Abstract] OR "patient activation"[Title/Abstract] OR "patients activation"[Title/Abstract] OR "patient engagement"[Title/Abstract] OR "patients engagement"[Title/Abstract] OR "patient empowerment"[Title/Abstract] OR "patients empowerment"[Title/Abstract] OR "patient comprehens*"[Title/Abstract] OR "patients comprehens*"[Title/Abstract] OR "patient satisfaction"[Title/Abstract] OR "patients satisfaction"[Title/Abstract] OR "experience*"[Title/Abstract] OR "understand*"[Title/Abstract] OR "interpret*"[Title/Abstract] OR "self-efficacy"[Title/Abstract]) | Result # 2,806 |

| **Search strategy in Web of Science (1,856 hits)** | | |
| --- | --- | --- |
| Search #1 | TS= "clinical laboratory service*" OR TS= "laboratory" OR TS= "laboratory test*" OR TS= "blood test*" OR TS= "test*" | Result # 7,215,906 |
| Search #2 | TS= "result*" OR TS= "outcome*" OR TS= "report*" | Result # 24,897,256 |
| Search #3 | TS= "health record*" OR TS= "electronic health record*" OR TS= "personal health record*" OR TS= "online health record*" OR TS= "patient portal*" OR TS= "electronic patient portal*" OR TS= "online patient portal*" OR TS= "patient access to records" OR TS= "computerized medical record*" | Result # 38,557 |
| Search #4 | TS= "patient education" OR TS= "education of patients" OR TS= "health literacy" OR TS= "health engagement" OR TS= "patient participation" OR TS= "patients participation" OR TS= "patient involvement" OR TS= "patients involvement" OR TS= "patient activation" OR TS= "patients activation" OR TS= "patient engagement" OR TS= "patients engagement" OR TS= "patient empowerment" OR TS= "patients empowerment" OR TS= "patient comprehens*" OR TS= "patients comprehens*" OR TS= "patient satisfaction" OR TS= "patients satisfaction" OR TS= "experience*" OR TS= "understand*" OR TS= "interpret*" OR TS= "self-efficacy" | Result # 5,864,623 |
| Search #5 | **Search #1 AND Search #2**  (TS= "clinical laboratory service*" OR TS= "laboratory" OR TS= "laboratory test*" OR TS= "blood test*" OR TS= "test*") AND (TS= "result*" OR TS= "outcome*" OR TS= "report*") | Result # 4,488,357 |
| Search #6 | **Search #5 AND Search #3**  (TS= "clinical laboratory service*" OR TS= "laboratory" OR TS= "laboratory test*" OR TS= "blood test*" OR TS= "test*") AND (TS= "result*" OR TS= "outcome*" OR TS= "report*") AND (TS= "health record*" OR TS= "electronic health record*" OR TS= "personal health record*" OR TS= "online health record*" OR TS= "patient portal*" OR TS= "electronic patient portal*" OR TS= "online patient portal*" OR TS= "patient access to records" OR TS= "computerized medical record*") | Result # 6,774 |
| Search #7 | **Search #6 AND Search #4**  (TS= "clinical laboratory service*" OR TS= "laboratory" OR TS= "laboratory test*" OR TS= "blood test*" OR TS= "test*") AND (TS= "result*" OR TS= "outcome*" OR TS= "report*") AND (TS= "health record*" OR TS= "electronic health record*" OR TS= "personal health record*" OR TS= "online health record*" OR TS= "patient portal*" OR TS= "electronic patient portal*" OR TS= "online patient portal*" OR TS= "patient access to records" OR TS= "computerized medical record*") AND (TS= "patient education" OR TS= "education of patients" OR TS= "health literacy" OR TS= "health engagement" OR TS= "patient participation" OR TS= "patients participation" OR TS= "patient involvement" OR TS= "patients involvement" OR TS= "patient activation" OR TS= "patients activation" OR TS= "patient engagement" OR TS= "patients engagement" OR TS= "patient empowerment" OR TS= "patients empowerment" OR TS= "patient comprehens*" OR TS= "patients comprehens*" OR TS= "patient satisfaction" OR TS= "patients satisfaction" OR TS= "experience*" OR TS= "understand*" OR TS= "interpret*" OR TS= "self-efficacy") | Result # 1,856 |

| **Search strategy in EMBASE (868 hits)** | | |
| --- | --- | --- |
| Search #1 | exp laboratory technique/ OR exp clinical laboratory service/ OR clinical laboratory service*.ti,ab,kf. OR laboratory.ti,ab,kf. OR laboratory test*.ti,ab,kf. OR blood test*.ti,ab,kf. OR test*.ti,ab,kf. | Result # 5,985,603 |
| Search #2 | result*.ti,ab,kf. OR outcome*.ti,ab,kf. OR report*.ti,ab,kf. | Result # 20,364,598 |
| Search #3 | exp electronic health record/ OR exp medical record/ OR health record*.ti,ab,kf. OR electronic health record*.ti,ab,kf. OR personal health record*.ti,ab,kf. OR online health record*.ti,ab,kf. OR patient portal*.ti,ab,kf. OR electronic patient portal*.ti,ab,kf. OR online patient portal*.ti,ab,kf. OR patient access to records.ti,ab,kf. OR computerized medical record*.ti,ab,kf. | Result # 339,939 |
| Search #4 | exp patient education/ OR exp health literacy/ OR exp patient participation/ OR patient education.ti,ab,kf. OR education of patients.ti,ab,kf. OR health literacy.ti,ab,kf. OR health engagement.ti,ab,kf. OR patient participation.ti,ab,kf. OR patients participation.ti,ab,kf. OR patient involvement.ti,ab,kf. OR patients involvement.ti,ab,kf. OR patient activation.ti,ab,kf. OR patients activation.ti,ab,kf. OR patient engagement.ti,ab,kf. OR patients engagement.ti,ab,kf. OR patient empowerment.ti,ab,kf. OR patients empowerment.ti,ab,kf. OR patient comprehens*.ti,ab,kf. OR patients comprehens*.ti,ab,kf. OR patient satisfaction.ti,ab,kf. OR patients satisfaction.ti,ab,kf. OR experience.ti,ab,kf. OR understand*.ti,ab,kf. OR interpret*.ti,ab,kf. OR self-efficacy.ti,ab,kf. | Result # 3,767,402 |
| Search #5 | **Search #1 AND Search #2**  (exp laboratory technique/ OR exp clinical laboratory service/ OR clinical laboratory service*.ti,ab,kf. OR laboratory.ti,ab,kf. OR laboratory test*.ti,ab,kf. OR blood test*.ti,ab,kf. OR test*.ti,ab,kf.) AND (result*.ti,ab,kf. OR outcome*.ti,ab,kf. OR report*.ti,ab,kf.) | Result # 4,323,195 |
| Search #6 | **Search #5 AND Search #3**  (exp laboratory technique/ OR exp clinical laboratory service/ OR clinical laboratory service*.ti,ab,kf. OR laboratory.ti,ab,kf. OR laboratory test*.ti,ab,kf. OR blood test*.ti,ab,kf. OR test*.ti,ab,kf.) AND (result*.ti,ab,kf. OR outcome*.ti,ab,kf. OR report*.ti,ab,kf.) AND (exp electronic health record/ OR exp medical record/ OR health record*.ti,ab,kf. OR electronic health record*.ti,ab,kf. OR personal health record*.ti,ab,kf. OR online health record*.ti,ab,kf. OR patient portal*.ti,ab,kf. OR electronic patient portal*.ti,ab,kf. OR online patient portal*.ti,ab,kf. OR patient access to records.ti,ab,kf. OR computerized medical record*.ti,ab,kf.) | Result # 69,905 |
| Search #7 | **Search #6 AND Search #4**  (exp laboratory technique/ OR exp clinical laboratory service/ OR clinical laboratory service*.ti,ab,kf. OR laboratory.ti,ab,kf. OR laboratory test*.ti,ab,kf. OR blood test*.ti,ab,kf. OR test*.ti,ab,kf.) AND (result*.ti,ab,kf. OR outcome*.ti,ab,kf. OR report*.ti,ab,kf.) AND (exp electronic health record/ OR exp medical record/ OR health record*.ti,ab,kf. OR electronic health record*.ti,ab,kf. OR personal health record*.ti,ab,kf. OR online health record*.ti,ab,kf. OR patient portal*.ti,ab,kf. OR electronic patient portal*.ti,ab,kf. OR online patient portal*.ti,ab,kf. OR patient access to records.ti,ab,kf. OR computerized medical record*.ti,ab,kf.) AND (exp patient education/ OR exp health literacy/ OR exp patient participation/ OR patient education.ti,ab,kf. OR education of patients.ti,ab,kf. OR health literacy.ti,ab,kf. OR health engagement.ti,ab,kf. OR patient participation.ti,ab,kf. OR patients participation.ti,ab,kf. OR patient involvement.ti,ab,kf. OR patients involvement.ti,ab,kf. OR patient activation.ti,ab,kf. OR patients activation.ti,ab,kf. OR patient engagement.ti,ab,kf. OR patients engagement.ti,ab,kf. OR patient empowerment.ti,ab,kf. OR patients empowerment.ti,ab,kf. OR patient comprehens*.ti,ab,kf. OR patients comprehens*.ti,ab,kf. OR patient satisfaction.ti,ab,kf. OR patients satisfaction.ti,ab,kf. OR experience.ti,ab,kf. OR understand*.ti,ab,kf. OR interpret*.ti,ab,kf. OR self-efficacy.ti,ab,kf.) | Result # 12,416 |
| Search #8 | **Search #7**  Filters: remove preprint records AND exclude MEDLINE citations | Result # 868 |
