# Supplementary material for: Enhancing Patient Understanding of Laboratory Test Results: Systematic Review of Presentation Formats and Their Impact on Perception, Decision, Action, and Memory
Source: J Med Internet Res. 2024 Aug 12;26:e53993. doi: 10.2196/53993 (PMC11347896; doi:10.2196/53993)
Supplement: Multimedia Appendix 3 [file jmir_v26i1e53993_app3.docx]

| Author (year) | Presentation format | | | | | | | | | | | | |
| --- | --- | --- | --- | --- | --- | --- | --- | --- | --- | --- | --- | --- | --- |
|  | Numerical | | Horizontal line bar | | | | | | Graph | | Video | Text only | Other |
|  | Reference range | Evaluative label | Colored blocks | Gradient coloring | Reference range | Evaluative label | Goal range | Harm anchors | Line graph | Triangle graph |  |  |  |
|  |  |  |  |  |  |  |  |  |  |  |  |  |  |
| Bar-Lev (2020) | x |  |  |  |  |  |  |  | x |  |  | x |  |
| Brewer (2012) | x |  | x |  |  |  |  |  |  |  |  |  |  |
| Elder (2012) | x | x | x |  |  |  |  |  | x | x |  | x |  |
| Fraccaro (2018) | x |  | x |  |  |  |  |  |  |  |  |  | Grouped presentation |
| Hohenstein (2018) | x | x |  |  | x |  |  |  |  |  |  |  |  |
| Kelman (2016) | x ^a^ | x ^a^ |  |  |  |  |  |  |  |  |  |  |  |
| Morrow (2017) |  |  |  |  |  |  |  |  |  |  | x |  |  |
| Morrow (2019) | x  x ^a^ | x ^a^ | x |  |  |  |  |  |  |  | x |  |  |
| Nystrom (2018) |  |  | x |  |  |  |  |  |  |  |  |  |  |
| Scherer (2018) | x |  | x |  |  |  | x |  |  |  |  |  |  |
| Struikman (2020) | x |  | x ^b^ |  |  | x ^b^ |  |  |  |  |  |  |  |
| Talboom-Kamp (2020) |  |  | x ^b^ |  |  | x ^b^ |  |  |  |  |  |  |  |
| Tao (2018) |  |  | x  x ^b^  x ^c^ |  | x | x ^b^  x ^c^ |  |  |  |  |  |  | Personalized information^c^ |
| Zarcadoolas (2013) | x | x |  |  |  |  |  |  |  |  |  |  |  |
| Zhang (2020) | x |  |  |  |  |  |  |  |  |  |  |  |  |
| Zhang (2021) |  |  | x ^d^ |  |  | x ^d^ |  | x ^d^ |  |  |  |  |  |
| Zikmund-Fisher (2017) | x |  | x | x | x |  |  |  |  |  |  |  |  |
| Zikmund-Fisher (2018) |  |  | x | x | x  x ^e^ |  |  | x ^e^ |  |  |  |  |  |

^a^Numerical display with both reference range and an evaluative label was combined into one studied variable.

^b^Horizontal line bars with both colored blocks and an evaluative label were combined into one studied variable.

^c^Horizontal line bars with colored blocks and personalized evaluative labels were combined into one studied variable.

^d^Horizontal line bars with colored blocks, an evaluative label and harm anchors were combined into one studied variable.

^e^Horizontal line bars with both reference ranges and harm anchors were combined into one studied variable.
