# Supplementary material for: Enhancing Patient Understanding of Laboratory Test Results: Systematic Review of Presentation Formats and Their Impact on Perception, Decision, Action, and Memory
Source: J Med Internet Res. 2024 Aug 12;26:e53993. doi: 10.2196/53993 (PMC11347896; doi:10.2196/53993)
Supplement: Multimedia Appendix 4 [file jmir_v26i1e53993_app4.docx]

| Author (year) | Score | Criteria from the Mixed Methods Appraisal Tool | | | | | | | | | | | | | | | | | | | | | | | | | | |
| --- | --- | --- | --- | --- | --- | --- | --- | --- | --- | --- | --- | --- | --- | --- | --- | --- | --- | --- | --- | --- | --- | --- | --- | --- | --- | --- | --- | --- |
|  |  | S1 | S2 | 1.1 | 1.2 | 1.3 | 1.4 | 1.5 | 2.1 | 2.2 | 2.3 | 2.4 | 2.5 | 3.1 | 3.2 | 3.3 | 3.4 | 3.5 | 4.1 | 4.2 | 4.3 | 4.4 | 4.5 | 5.1 | 5.2 | 5.3 | 5.4 | 5.5 |
|  |  |  |  |  |  |  |  |  |  |  |  |  |  |  |  |  |  |  |  |  |  |  |  |  |  |  |  |  |
| Bar-Lev et al. (2020) | *** | Y | Y |  |  |  |  |  |  |  |  |  |  |  |  |  |  |  | N | C | Y | Y | Y |  |  |  |  |  |
| Brewer et al. (2012) | * and ** | Y | Y |  |  |  |  |  | C | Y | C | C | C | N | Y | C | C | Y |  |  |  |  |  |  |  |  |  |  |
| Elder et al. (2012) | ***** | Y | Y | Y | Y | Y | Y | Y |  |  |  |  |  |  |  |  |  |  |  |  |  |  |  |  |  |  |  |  |
| Fraccaro et al. (2018) | **** | Y | Y |  |  |  |  |  |  |  |  |  |  | Y | Y | Y | C | Y |  |  |  |  |  |  |  |  |  |  |
| Hohenstein et al. (2018) | **** | Y | Y |  |  |  |  |  |  |  |  |  |  |  |  |  |  |  |  |  |  |  |  | Y | Y | C | Y | Y |
| Kelman et al. (2016) | ***** | Y | Y |  |  |  |  |  |  |  |  |  |  |  |  |  |  |  | Y | Y | Y | Y | Y |  |  |  |  |  |
| Morrow et al. (2017) | *** | Y | Y |  |  |  |  |  |  |  |  |  |  |  |  |  |  |  |  |  |  |  |  | C | Y | Y | Y | C |
| Morrow et al. (2019) | 0 | Y | Y |  |  |  |  |  | C | C | C | C | C |  |  |  |  |  |  |  |  |  |  |  |  |  |  |  |
| Nystrom et al. (2018) | **** | Y | Y |  |  |  |  |  |  |  |  |  |  |  |  |  |  |  |  |  |  |  |  | Y | Y | C | Y | Y |
| Scherer et al. (2018) | ** | Y | Y |  |  |  |  |  | C | C | Y | C | Y |  |  |  |  |  |  |  |  |  |  |  |  |  |  |  |
| Struikman et al. (2020) | **** | Y | Y |  |  |  |  |  | C | Y | Y | Y | Y |  |  |  |  |  |  |  |  |  |  |  |  |  |  |  |
| Talboom-Kamp et al. (2020) | *** | Y | Y |  |  |  |  |  |  |  |  |  |  |  |  |  |  |  | Y | C | Y | N | Y |  |  |  |  |  |
| Tao et al. (2018) | *** | Y | Y |  |  |  |  |  |  |  |  |  |  | C | Y | C | Y | Y |  |  |  |  |  |  |  |  |  |  |
| Zarcadoolas et al. (2013) | ***** | Y | Y | Y | Y | Y | Y | Y |  |  |  |  |  |  |  |  |  |  |  |  |  |  |  |  |  |  |  |  |
| Zhang et al. (2020) | **** | Y | Y |  |  |  |  |  |  |  |  |  |  |  |  |  |  |  |  |  |  |  |  | Y | Y | Y | Y | N |
| Zhang et al. (2021) | ***** | Y | Y |  |  |  |  |  |  |  |  |  |  |  |  |  |  |  |  |  |  |  |  | Y | Y | Y | Y | Y |
| Zikmund-Fisher et al. (2017) | ***** | Y | Y |  |  |  |  |  |  |  |  |  |  |  |  |  |  |  | Y | Y | Y | Y | Y |  |  |  |  |  |
| Zikmund-Fisher et al. (2018) | *** | Y | Y |  |  |  |  |  | Y | C | Y | C | Y |  |  |  |  |  | Y | Y | Y | Y | Y |  |  |  |  |  |

Abbreviations: Y, yes; N, no; C, can’t tell.

Score was calculated as follows: for each ‘Y’ 1 star was awarded, while ‘N’ or ‘C’ did not receive any stars.
